# Perceived impacts of a pilot agricultural livelihood and microfinance intervention on agricultural practices, food security and nutrition for Kenyans living with HIV

**DOI:** 10.1371/journal.pone.0278227

**Published:** 2022-12-14

**Authors:** Tammy M. Nicastro, Lauren Pincus, Elly Weke, Abigail M. Hatcher, Rachel L. Burger, Emiliano Lemus-Hufstedler, Elizabeth A. Bukusi, Craig R. Cohen, Sheri D. Weiser

**Affiliations:** 1 Department of Obstetrics, Gynecology & Reproductive Sciences, University of California, San Francisco, San Francisco, California, United States of America; 2 Independent Consultant, Washington, CT, United States of America; 3 Centre for Microbiology Research, Kenya Medical Research Institute, Nairobi, Kenya; 4 Gillings School of Public Health, University of North Carolina, Chapel Hill, North Carolina, United States of America; 5 School of Public Health, University of the Witwatersrand, Johannesburg, South Africa; 6 School of Medicine, University of California, San Francisco, San Francisco, California, United States of America; 7 Department of Global Health, University of Washington, Seattle, Washington, United States of America; 8 Department of Obstetrics and Gynecology, University of Washington, Seattle, Washington, United States of America; 9 Division of HIV, Infectious Diseases, and Global Medicine, Department of Medicine, University of California, San Francisco, San Francisco, California, United States of America; International Maize and Wheat Improvement Centre: Centro Internacional de Mejoramiento de Maiz y Trigo, MEXICO

## Abstract

**Introduction:**

Agriculture is the primary source of income and household food for >75% of rural Kenyans, including people living with HIV (PLHIV), making agricultural yields an important factor in food security and nutrition. Previous studies have shown the interconnectedness of food insecurity, malnutrition, and poor HIV health by elucidating that having one of these conditions increases the likelihood and severity of having another. However, few studies have explored the linkages between agricultural practices, food security and nutrition for PLHIV, or how agricultural livelihood interventions may affect these domains. This study aimed to examine the mechanisms through which an agricultural livelihood intervention can positively or negatively affect agricultural practices, food security, and nutrition for PLHIV.

**Methods:**

From July 2012-August 2013, we interviewed participants with HIV on antiretroviral therapy (ART) enrolled in a pilot randomized controlled trial (RCT) of an agricultural livelihood and finance intervention to understand the mechanisms through which the intervention may have affected HIV health outcomes. The intervention included agricultural and finance training and a microfinance loan to purchase the MoneyMaker hip pump, a human-powered water pump, seeds, and other farming implements. A purposive sample of 45 intervention and a random subset of 9 control participants were interviewed at 12-month endline visit with a subset of 31 intervention participants interviewed longitudinally at both the 3- and 12-month visits. Transcripts were double coded using an inductive-deductive approach and analyzed for impacts of the intervention on agricultural practices, food security, and nutrition using analytic reports for each key theme.

**Results:**

All intervention participants described improvements in agricultural practices and yields attributed to the intervention while many also described improvements in income; these changes in turn contributed to improved HIV health, including suppressed viral loads, and a few people noted improved immunologic parameters. Key mechanisms included the knowledge gained from agricultural training which led to improved yields and access to new markets. The use of the irrigation pump was also identified as an additional, lesser important mechanism. All intervention participants reported sustained improvements in food security and nutrition through increased yields and income from the sale of excess crops used to purchase food, and diversification of fresh fruits and vegetables consumed through agricultural production. This led to self-reported weight gain which was a nutritional mechanism towards improved health.

**Conclusions:**

Agricultural and finance interventions that improve farming practices could lead to improved health outcomes through the pathways of improved food security, income, and diversified diet. The results from this study helped the team to enhance the intervention prior to implementation of the larger cluster RCT (cRCT). By understanding how agricultural livelihood interventions act upon pathways towards improved health, policy options can be developed and implemented to include components that are needed to achieve sustainable outcomes.

**Trial registration:**

ClinicalTrials.gov NCT01548599.

## Introduction

Food security is defined through four dimensions: having enough food available, physical and economic access to enough food, food availability and accessibility that remains stable, and being able to biologically utilize food such that nutrients are absorbed [1]. Conversely, food insecurity occurs when any one of these dimensions is compromised and inadequate amounts of food are consumed and/or nutrients absorbed. Food insecurity, malnutrition, and HIV are tightly connected in a positive feedback loop, with each condition increasing the risk of and elevating the severity of the other [2–9] Food insecurity can cause more rapid progression of HIV infection, is associated with a higher rate of HIV transmission from mother to child, as well as increased mortality [2, 8, 10, 11]. Food insecurity impedes optimal adherence to antiretroviral therapy (ART) and clinic visits directly or indirectly and contributes to worse virologic and immunologic outcomes [6, 12]. Food insecurity has also been shown to increase the risk of HIV transmission because of the relationship between food insecurity and unsafe sexual practices which can be used as a means of securing food [2, 13]. Finally, food insecurity has been linked to stigma and depression separately in people living with HIV (PLHIV) [5, 14, 15]. Stigma towards PLHIV reinforces a breakdown in use of social networks and social protection systems, such as asking for food support from family or friends, which play an important role in food security in resource-limited settings (RLS) [5].

The proportion of people living in Kenya who experience food insecurity and suffer from malnutrition is roughly 36% [16, 17]. The Nyanza region has the country’s highest proportion of PLHIV (14%) [18], and 50–70% of PLHIV in this region experience food insecurity, placing this population among the most vulnerable in the region [16, 19–21]. In Kenya and globally, 80% of the poor derive their income and household food from smallholder farms [22–25]. Agriculture provides 26% of Kenya’s GDP and an additional 27% is generated indirectly by agricultural impact on other sectors [26] But despite the pervasive role of agriculture in Kenya, food insecurity remains a problem for many smallholder farmers and their families because of a lack of farming infrastructure and knowledge on effective farming techniques to meet current weather changes, required to produce adequate food to meet nutritional needs [27]. Growth in agriculture that results in GDP growth has been studied extensively for impacts on reducing poverty among the world’s poorest [28, 29] because farming is the primary source of income for 80% of this population [25]. A 2018 edition of World Development focused on agricultural development and poverty reduction including empirical studies across and within countries that employed econometrics and modeled simulations to estimate impacts of GDP growth in agriculture and non-agriculture sectors on poverty reduction [28, 30–32]. Recent studies have shown GDP growth in agriculture is two-three times more effective than GDP growth from any other sector at reducing poverty globally [28, 29]. Moreover, the literature indicates that the poorest people experience the greatest difference between agriculture and non-agriculture sector GDP growth on poverty reduction [28, 29], thus strengthening the case for agricultural livelihood interventions when addressing the pathway between poverty and food insecurity for rural populations [29, 33].

Agricultural interventions present an opportunity to affect poverty and food insecurity at the household level through changes in agricultural outputs that lead to increased income and purchasing power, which ultimately can lead to improvements in HIV health. Multiple studies have been conducted showing the positive impact of agricultural interventions on income and food security, [34–36] however little has been published on the impacts of such interventions on nutrition among PLHIV, or the mechanisms towards improvements. The objective of this study was to examine the mechanisms through which an agricultural livelihood intervention may affect agriculture practices, food security, and nutrition for PLHIV. These findings can fill a gap in the literature by describing pathways outside traditional healthcare, that PLHIV depend upon for food security and nutrition, both of which impact HIV health outcomes [3–9, 37].

## Methods

**Fig 1:**
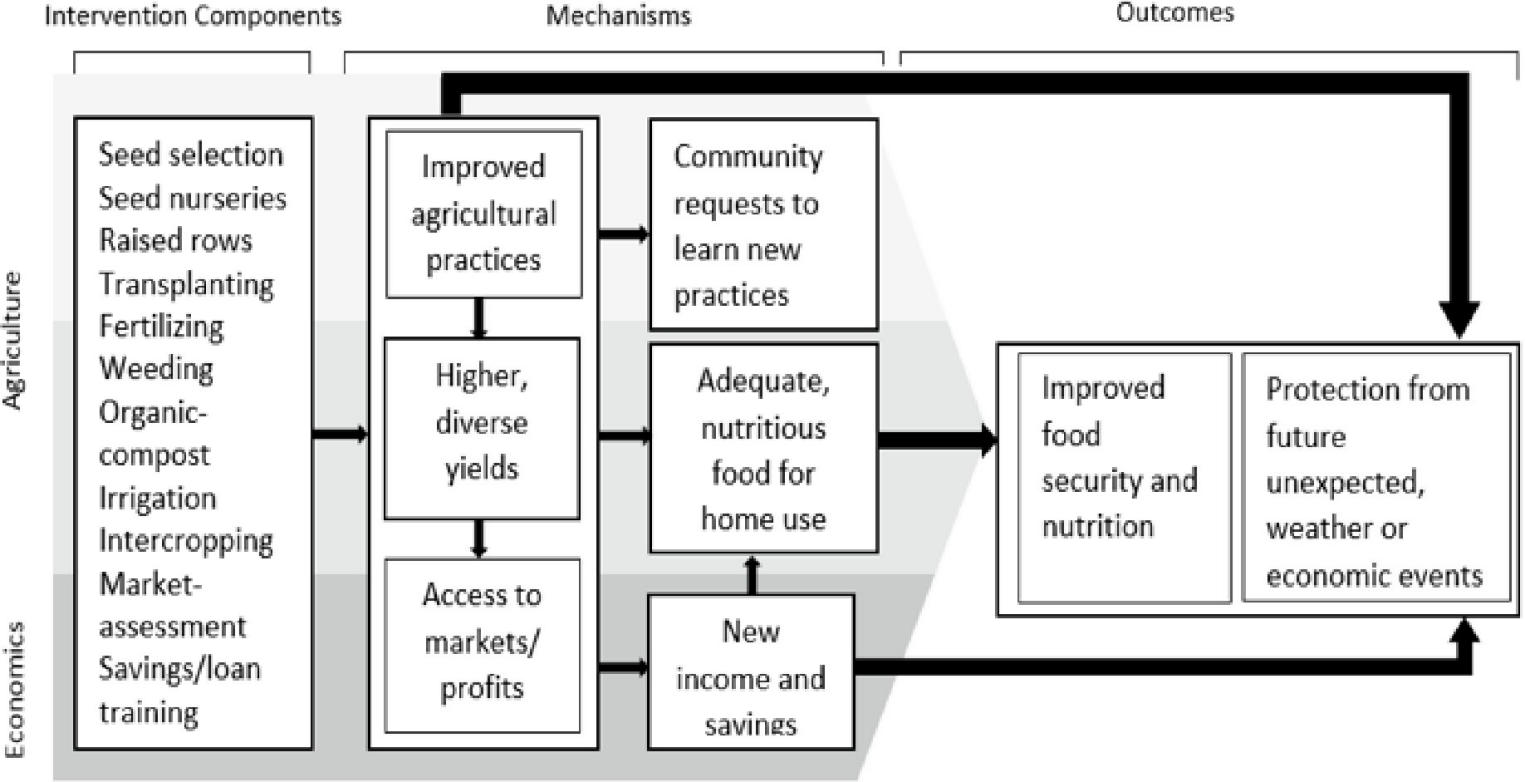
Conceptual framework of mechanisms between a multisector agricultural intervention and improved food security, nutrition, and protection from future shocks.

This longitudinal qualitative study was conducted within a pilot study titled Shamba Maisha (NCT01548599), which was a randomized control trial (RCT) using a multisectoral agriculture and finance intervention to improve HIV health outcomes among PLHIV in Migori county in Kenya [38]. The pilot study aimed to test whether a multisectoral agricultural and finance intervention improves food security and HIV outcomes, and to test possible mediating pathways with mixed methods using a conceptual framework. Agriculture was identified as a health determinant in this framework with food security and nutritional status as mediating factors, however it was unknown exactly how agriculture could influence food security and nutrition among PLHIV. Additionally, it was unknown how inclusion of the MoneyMaker hip pump in the agriculture intervention would be utilized by PLHIV to address persistent dry conditions. In this qualitative study, we focus on participant perceptions of impacts of the agricultural livelihood intervention on agricultural productivity, food security and nutrition using the conceptual framework depicted in Fig 1. This study design allowed us to further evaluate the process evaluation data [38] to understand which changes, if any, should be made to improve the subsequent cluster randomized control trial (cRCT), while preserving the components shown to be on causal pathways towards any improved outcomes.

The agricultural training as previously described [38] included the following components: use of the MoneyMaker hip irrigation pump, instructional and practical trainings on seed selection, sustainable soil and water management, sustainable methods for crop rotation, fertilization, pest and disease management, and crop handling and marketing both pre- and post-harvest. Training to identify optimal market access for crops grown was integrated into farmer training that included participants conducting surveys of local markets to identify demands for certain crops. Finally, participants received financial training on establishing savings accounts, the components of a loan, record keeping and group dynamics.

The quantitative findings of the pilot study showed viral suppression was seven times more likely in the intervention arm as compared to the control arm (odds ratio 7.6, 95% confidence interval 2.2–26) [39]. Study design was not informed by the quantitative findings, since both quantitative and qualitative studies took place synchronously by different research teams [38]. We identified four mediating pathways: nutrition, empowerment, adherence, and mental health and have published on each of the other pathways other than nutrition [13, 40–42]. We designed this qualitative study to understand the mechanisms between the pilot agricultural livelihood intervention and improvements in food security and nutrition. Control participants were offered intervention components upon completion of the study. Both arms had access to the same HIV care from local health facilities.

### Participants

Participants were identified from two health facilities after expressing interest in the study. Screening was conducted and if eligible, participants provided written consent. Inclusion criteria for the parent study were as follows: PLHIV who were farmers between the ages of 18–49 living in the Rongo and Migori districts in Nyanza Province, Kenya, on ART, had access to farmland and surface water and demonstrated evidence of moderate to severe food insecurity or malnutrition (BMI <18.5) during the year preceding the study. A full description of the study subject inclusion criteria and recruitment procedures can be found elsewhere [38].

### Data collection

The qualitative study data were collected from July 2012-August 2013 and included interviews of intervention (n = 45) and control (n = 9) participants who were selected using a purposive method to establish a sample reflective of the gender and age of the larger Pilot Shamba Maisha cohort. Interviews were conducted to determine if intervention participants’ experiences related to impacts from the intervention were due to intervention components or participation in the study. We followed some intervention (n = 31) participants longitudinally with an interview at month 3–5 post-enrollment and again at intervention end, 12 months post-enrollment. Control participants were only interviewed at month 12.

Data were collected through structured interviews at participants’ homes, farms, and health facilities during the pilot study. Interviews were conducted by trained research staff using a structured guide and focused on changes in agricultural practices, yields, income, food insecurity and health that may have occurred in general over the course of the study for both intervention and control participants. For intervention participants, we included additional questions and probes to reflect on what worked and did not work with the different intervention components and how the intervention may have impacted agricultural practices, yields, food security and nutrition. Interviews were digitally recorded and transcribed by the research assistant who conducted the interview. Interviews typically lasted 45 minutes to 2 hours. Research Assistants conducted the interviews in the region’s native language or in English. Participants were compensated 400–500 Kenyan Shillings (3.50–4.50 USD) for their time. The study was approved by the Committee on Human Research at the University of California San Francisco (UCSF) and the Ethical Review Committee at the Kenya Medical Research Institute (KEMRI). The clinical trial was registered at ClinicalTrials.gov (NCT01548599).

### Analysis

During the parent study, transcripts were translated verbatim using emic words and phrases to retain local meanings that otherwise would have been lost in translation. Translation accuracy was managed by a review process post-translation that involved a study investigator who would in turn relay any questions to the interviewer. Transcripts were reviewed by an investigator confirming the translation was clearly understood and providing feedback to research assistants regarding responses that would benefit from probing for future interviews. Each transcript was labeled with location, age, time point, and participant gender. Transcripts were managed using Dedoose coding software.

We used a framework analysis with an integrated inductive-deductive process to conduct this analysis. First, a random sample of transcripts was reviewed, and analytic memos written to capture themes or issues arising in the context of agriculture, food security, and nutrition. These initial findings were discussed among the study team and evaluated for emergent themes. Next, a broad thematic coding framework was established using our hypotheses related to food security, agriculture, and nutrition, as the first step in the deductive analysis. We also included broad themes that emerged from the memo process that were not previously conceptualized, as the first step in our inductive analysis. Throughout the coding process we created new codes that emerged through the inductive reasoning approach. Finally, we established fine codes that emerged through the analysis process. All transcripts used for this analysis were double coded. After all coding was complete, we created an analytic framework table to synthesize key findings and identify relevant quotations and excerpts.

### Inclusivity in global research

Additional information regarding the ethical, cultural, and scientific considerations specific to inclusivity in global research is included in the Supporting Information (S1 Checklist. Inclusivity in global research.).

## Results

### Demographic characteristics

A subset of 54 HIV-positive participants from the Shamba Maisha parent study were included in the parent study qualitative cohort. This cohort was comprised almost equally of men and women ranging in age from 23–56 years, almost all (96%) were parents of at least two children (Table 1). Participants who were married (60%) fell into two marriage categories: monogamous (20) or polygamous (11) with one husband and multiple wives. All other participants were widows (21) either living as the sole adult in the household or they were living with family members of their deceased husband, a practice commonly known in this region as being inherited. Participants’ experience with ART varied, but most (86%) had 2 or more years of ART before the start of the study. All participants had experience farming with a median of 13 years.

**Table 1:** Participant socio-demographics.

Participant Group	Participants (n = 54) Number (%) or median (IQR)
Intervention (longitudinal)	31 (57)
Intervention (endline)	14 (26)
Control (endline)	9 (17)
Male	28 (52)
Female	26 (48)
Median age	38 (33–42)
Median years on ART	4 (2–6)
Married (monogamous)	20 (38.5)
Married (polygamous)	11 (21.2)
Widow (single)	15 (28.8)
Widow (inherited)	6 (11.5)
Median number of children	3 (2–4)
Median years farming	13 (5–20)

Types of prior farming experience varied from small kitchen gardens used primarily for home consumption, to kitchen gardens plus cash crop farming such as maize or sugar cane. In addition, it was common for participants to have prior experience raising livestock either for home use of dairy and poultry or in commercial settings. A few had experiences as fishermen, while others had experience with commodity crops such as cotton or tobacco. More than a quarter of participants had other farmland that was not used for this study’s evaluation, and they continued farming on this land during the study period while concurrently farming their crops designated for study purposes. Lastly, less than 1/3 of participants had experience as paid farm workers for larger farms while a few had experiences employing farm workers on their land.

### Perceived impacts of the intervention on agricultural yields and practices

Participants described two primary changes in yields in their farming since joining Shamba Maisha: larger amounts of crops harvested and more diversified crops with more nutritional value than yields prior to Shamba Maisha. Improvements in yields were a result of three connected mechanisms described as 1) changes in farming practices 2) use of the irrigation pump, and 3) access to new markets which drove plans for seed selection and plot size to meet new selling opportunities. Control participants did not report experiencing any changes in their agricultural yields or practices and instead reported ongoing concerns like failed crops.

#### Mechanism 1: Changes in farming practices that led to improved yields

Intervention participants noted several changes in their farming practices because of Shamba Maisha, including expansion in their plot size, seed selection and nurseries, and new practices such as implementing raised rows/ridges, the use of fertilizers, and the practice of weeding. One male participant described changes in planting and pest control that allowed him to farm as a trained agriculturist:

“Before Shamba Maisha I used to plant randomly without giving much attention to what I am planting. I would not even know the number of seeds I have planted. However, when one cultivates like an agriculturist you must know how many seeds you cultivate in each line, and you can even prevent pests from destroying your crops. That is how I have changed from my previous planting practices where I used to plant ignorantly. Ever since I was trained, I now plant as someone who is agriculturally sound.” (Male participant, intervention end, 36 years old)

All intervention participants described using new farming techniques addressing the handling and planting of seedlings: As described by a 30-year-old female intervention participant:

“I have experienced a lot of changes as before we just cultivated our crops but now, we use ridges which we learnt through Shamba Maisha.”

Another female participant described improved yields which she attributed to the intervention training on seed nurseries and transplanting:

“We used to not cultivate the vegetables in a line, they were scattered all over and no nursery was prepared for the seeds. That is something that caused our yield to be low. Currently, we have a new way of plucking (transplanting) the vegetables that takes more time before they grow other leaves and takes longer in the farm but produces more yield…I was taught how to plant vegetables like the black night shade and kale when I joined Shamba Maisha and now, I have stopped planting them the traditional way. I have mastered how to separate at least two units of kale and I now plant the seedlings in a nursery. That is something that I did not use to do.” (Female participant, intervention end, 35 years old)

“Yes, I have seen changes in the workload. I now cultivate only a small piece of land and get good yield, unlike the other times (before Shamba Maisha) that I used to cultivate a large tract of land.” (Female participant, intervention end, 35 years old)

A 44-year-old male participant who used to farm without raised beds, changed his method to incorporate raised beds and as a result was also able to reduce the overall size of his plot:

“…when Shamba Maisha came, they told us that every crop must be placed on a raised bed. Before we could just spread the seeds randomly as we still do with maize now, but we realized that we were using a bigger space compared to this new method of putting them on raised beds. What I am seeing is that it (the farm) can produce more compared to before because it is more fertile. I use a small area and even the wasted plant materials that I get from there are spread over the farm which then decomposes and increasing the fertility of the soil.”

#### Mechanism 2: Use of the irrigation pump that led to improved yields

Utilization of the irrigation pump during droughts was a less pronounced theme, but some participants attributed their improvements in agricultural practices to the use of the pump to keep crops growing during droughts as well as to capitalize on market opportunities that most farmers without irrigation pumps would not be able to do. Importantly, while the irrigation pump was useful for drought conditions, it did not help participants during their farming during periods of excessive rain. One participant described the nuances of this as follows:

“It (farming) is generally good; however, the weather hasn’t been favorable for most of our crops that we cultivated by the riverbeds. It has been raining too much lately, although now we have been experiencing a drought. We managed to use the water pumps, which turned out to be very effective.”(Female participant, intervention end, 35 years old)

Another participant described how the irrigation pump enabled him to capitalize on a market for sukuma (collard greens):

“We have experienced the dry season for a long duration now and so many people lacked sukuma, but I was able to pump water, so many people from different places were coming to buy them from me. That is how I became famous; people used to spread the word that there is someone out here who has and sells sukuma and they would give out my name. They would say that if you go to me, you will find sukuma and when they paid me a visit, they never were missing vegetables. That’s how I got famous!” (Male participant, intervention end, 36 years old)

This participant attributed improvements in her farming and income to the irrigation pump:

“It has brought some change since I have been taught how to farm and even when there is drought, I am able to get vegetables, which give me a source of income that I didn’t use to have.” (Female participant, intervention end, 35-year-old)

#### Mechanism 3: Access to new markets that led to improved yields

Access to new markets as a result of increased yields was a salient theme that emerged throughout the interviews. Five types of markets were described by participants: 1) home-farm markets, 2) produce markets open to the public, 3) passersby asking to buy vegetables while participants walked to produce markets, 4) hospitals and clinics where staff request produce to purchase for individual consumption, and 5) schools, grocery stores, or hotels, for large-scale food production. If participants pooled their yields with other members of their farmer groups in Shamba Maisha, they could supply customers with large orders that they would not have access to as an individual farmer, as a 42-year-old intervention male participant describes:

“Yes, if we put our hands together then we are able to do that (supply a large order); we can look for one market, put our produce in one market and supply it as a group.”

One male intervention participant describes the passersby market opportunity, which occurs commonly in this setting when a farmer has fruit or vegetable that is typically difficult to find at a market and they are repeatedly stopped by passersby who offer to buy their crop:

“As I may be harvesting to take my vegetables to a certain market and I get a passerby who wants to buy them, I sell to him. I sell readily on the way to anyone who wants to buy my crops and sometimes I exhaust them even before reaching my intended destination. Marketing is very easy unlike the planting.” (Female participant, intervention end, 30 years old)

A key theme expressed by almost all participants who previously kept only a kitchen garden for home consumption, was how the farmer training provided skills that empowered them to grow crops to sell. All participants described how earning an income from their farms enabled them to purchase items for their home, pay their children’s school fees, pay for transportation to the HIV clinic, and purchase diverse food. One participant described changes in ability to utilize farming as a source of income with some degree of surprise at how much demand existed for their crops, a female intervention participant said:

“In the past my income was low, so I used to struggle a lot and still I was not able to get enough food for my children. But currently my children have plenty of fruit as well as vegetables that they can pick and eat. At times, customers come, and we even sell vegetables when I am not around so the family can buy things that we don’t have in the house.”

A female intervention participant describes how the agricultural livelihood training taught her how to generate a consistent income from farming instead of farming only for household consumption:

I never used to plant vegetables for sale. I just used to plant it only for my family, but Shamba Maisha taught me about that. For example, right now, the indigenous vegetable that I planted I get people coming every evening wanting it for Kshs (Kenyan Shillings) 50, 100… (Female participant, intervention end, 32 years old)

Participants also described the use of a middleperson to either bring their crops to market or allow them access to certain market opportunities:

“Well, it has been good because I have had enough vegetables for my family and maybe some little that I can supply the local vegetable vending women to take to the market. There was also a time I took the produce from my first harvest to Kanyawanga School through someone.” (Male participant, intervention end, 39 years old)

There were also lost market opportunities that participants described either due to weather damaging crops or participants not knowing how to predict market saturation or shortages. One 32-year-old, female intervention participant said:

“Because sukuma wiki (collard greens) was destroyed very much by the rain and there is none in the market, if I had sukuma wiki now, like I did the last season, I would really have so much money that I wouldn’t know what to do with it.”

While another participant described a lost market opportunity while going through a period with very high demand for vegetables but not enough supply:

“Right now people are coming, and I am turning them away because I can’t supply all of them; in fact, I have just realized that I should have made the farm much bigger than it was.” (Male participant, intervention end, 42 years old)

Generally, most participants had access to markets because their crops were meeting a demand that exceeded the supply in most parts of the region. A 44-year-old male participant explains:

“Finding a market is not really a problem now because produce like vegetables are on high demand due to the shortage caused by the rains. So, if we have produce, there would be a market for them, and it would not really be a problem.”

### Multiple mechanisms interconnected

The interconnectedness of the three mechanisms that led to improved agricultural yields and practices was commonly reported in intervention participant responses. These responses provide additional insights into the ways in which improved outcomes were reliant upon utilizing multiple aspects of the agricultural training.

A 35-year-old female intervention participant describes how the use of connecting mechanism 1 and 3 taught her to use intercropping as a means of expanding and commercializing her farming practices:

“I used to plant vegetables for mere subsistence, but I have now learned to plant them for commercial purposes, so I have set aside a bigger portion of farmland for it. We now practice intercropping whereby we plant maize seasonally in the same farm and mix it with beans and maybe bananas or beans at the same time.”

Another 35-year-old female participant describes her new ability to expand her plots and earn an income because of her “plentiful” yields and the support of the Shamba Maisha team:

“…after I have harvested say maize, I sell some and get money in case it is plentiful. Again, I am able to sell vegetables. They have really encouraged me and that is why I have acquired another piece of land for farming.”

She goes on to describe how she is using a different farming technique than the previous participant, illustrating how many farmers were able to utilize guidance on farming techniques optimized for their specific farm:

“I am using it (the new piece of land) only for vegetables while the other one was just for maize and indigenous vegetables called bo.”

Several participants commented on how the agricultural training decreased their workload, giving them more time for other competing demands for time or energy. One female participant describes how the intervention has optimized her labor, reducing the size of land required, and thereby reducing the amount of work required to manage the crops, to achieve a good yield.

### Perceived impacts of the intervention on food security and nutrition

A major theme expressed throughout all the intervention participant interviews was the positive impact the intervention had on food security and nutritional status because of the mechanisms described above, which led to improved yields and income. Three additional mechanisms or subthemes were identified through participants’ description of ways improved yields led to improved food security and nutrition: 1) more nutritious food was available for home consumption; 2) income from the sale of excess crops was used to supplement the diet and diversify it; and 3) participants described how intervention training on diversified seed selection for a balanced diet, improved their food security and nutrition. Control participant interviews reflected a continued state of food insecurity consistent with their food insecurity status upon entering the study which made them eligible for study participation.

#### Mechanism 1: More nutritious food available for home consumption

A common theme was having more nutritious foods from the farms for home consumption. One 32-year-old female participant expressed a substantial change in food security early in the study, at month three describing the ways improved yields impacted food security and nutrition, she stated:

“One of the changes that I can say is that, at this time of the year you would normally find that there is no food in the farms and people have to go to the markets, but instead of doing that, now you can have enough vegetable in the farm you can just pick them…”

Another 32-year-old female participant who prior to participating in Shamba Maisha often went without regular meals for a week at a time, described how after joining Shamba Maisha she has not experienced any food insecurity:

“Since I joined Shamba Maisha, my kids have never gone without food, but before joining Shamba Maisha there are times when I could have totally nothing to eat. This is no longer the case.”

Another participant, who described herself as not being a good vegetable farmer before Shamba Maisha, would routinely have to buy vegetables from the nearest market in Rongo. However, after joining Shamba Maisha she describes a steady supply of vegetables she grows as well as opportunities to diversify her diet:

“Currently I can do without buying vegetables even with heavy rains like the ones we recently experienced. Even now as we talk, I have access to vegetables which I can consume. Since I joined Shamba Maisha one year ago, I have never gone to Rongo market to buy vegetables. The money I used before to buy vegetables is now channeled to buy tomatoes or small fish. (Female participant, intervention end, 30 years old)

Many participants described major changes in their diet which contributed to increases in energy levels. A 33-year-old female participant who previously experienced several hospitalizations said about her nutritional experience after joining Shamba Maisha:

“We are able to eat well, and I am more energetic since I joined Shamba Maisha. The vegetables which we were encouraged to eat have helped me and I have never been bed ridden since I joined Shamba Maisha.”

This participant described how she used to buy most vegetables she fed her family and she never had enough to feed everyone; people were always hungry. After joining Shamba Maisha, she described a food secure household consuming double the value of what she used to purchase:

“…but now the vegetables that I have no matter how little or bad they are, we pick them and cook them here in our house very well and we get satisfied. The vegetables that I used to buy was so little and could not satisfy my family well but the ones that I have planted by myself I find that I have planted something like ‘osuga’ (indigenous vegetables) when I go pick some it satisfies my family so much. …(the food we used to eat) was little, say if I bought for Kshs 50 it was put very little that was not enough for me. But now when I go pick what we are supposed to eat from my farm and compare it to what I used to buy for Kshs 50, I see that it could even be worth Kshs 100, and everybody is satisfied. (Female participant, intervention end, 28-year-old)

#### Mechanism 2: Income from sale of excess crops used to supplement and diversify diet

Improved income from surplus yield sales was a major theme expressed by all intervention participants as a key mechanism towards improved food security and nutrition. A 32-year-old female participant states:

“I can sell vegetables and use the money to buy omena (small fish), flour, and other foodstuff. So, the vegetables become the source of other foods. I can sell it and buy grains like people are doing now… so if it was not there, I don’t know what would happen.”

A mother who previously lacked the income to buy necessities for her baby noted this change after joining Shamba Maisha:

“There is a change because I am able to buy milk for my baby and pay every end of the month (bill) because I have the money from Shamba Maisha.” (Female participant, intervention end, 30-year-old)

Another mother who described previously being unable to provide for her children said because of Shamba Maisha she was able to not only provide food for her children but provide a diversified diet:

“This is because I was advised to plant various types of vegetables so that even if I have no money, I can sell vegetables and get money to buy proteins and carbohydrates. So currently I have no challenge in terms of getting food. I am doing well.” (Female participant, intervention end, 35-year-old).

Similarly, a male who previously struggled to provide food for his family describes an example of food security and improved nutrition because of Shamba Maisha:

“Right now the food I have is enough for my family because the money I get from selling sukuma I normally use it to buy meat, fish, or small fish… and also vegetables, because I must look for a balanced diet too. Since I joined the group, nowadays I see I have more money but when I was not part of the group, I would not even have 10 shillings in one week. However, currently I normally get Kshs. 200 or even Kshs. 500 in a week.” (Male participant, intervention end, 36-year-old)

Intervention participants commonly referred to their nutritional intake before Shamba Maisha as being poor, while attributing newly experienced diversified diets to Shamba Maisha training:

“Before, I did not know much but Shamba Maisha has enabled me to consume different kinds of food.” (Female participant, intervention end, 30-year-old)

#### Mechanism 3: Training on diversified seed selection for balanced diet

A salient theme throughout the interviews were participant perceptions that their and their children’s nutrition improved as a result of Shamba Maisha agricultural training on nutrient rich crops and seed selection to diversify crops and introduce new fruits and vegetables. Participants often commented on how their new food security and improved nutrition improved their HIV outcomes:

“The reason for the increase in CD4 [lab result] is that I have reduced stress and at the same time I am eating a balanced diet now that I have different types of vegetables from the farm. If someone has drug related side effects, it’s an indication of bad health. There are those who I know who were taking ARVs but were not eating well and died as a result of that. Someone on ARVs must just eat a well-balanced diet so that they live longer. For instance, we have vegetables in our farm and have also planted fruit like pawpaw, avocadoes, and bananas. These we get easily, and they are good supplements for the foods that we are eating.” (Female participant, intervention end, 35-year-old)

This participant referenced indigenous vegetables being consumed with protein to balance her nutritional intake as well as improving her nutrition through the replacement of potatoes from ugali to rice:

“Since I joined Shamba Maisha I find that I am able to balance my diet very well here in my family because you find that I have these different types of indigenous vegetables like ‘osuga’, ‘apoth’ and also ‘sukuma wiki’ so at times you find that have bought some meat and it’s taken together with ‘osuga’ that means am balancing my diet, the next day I can buy ‘omena’ and its eaten together with ‘apoth’…so you find that I am very ok and at times we stop eating ‘ugali’ and instead we eat rice…so I find that I can balance the diet well.” (Female participant, intervention end, 28 years old)

### Perceived impacts of the intervention on future protection from unexpected weather or economic events

One-fourth of intervention participants described protection from future unexpected weather or economic events, as a result of Shamba Maisha agricultural and finance training, as an emergent theme. Mechanisms of protection included: 1) confidence in skills with enduring value acquired through intervention training; 2) ownership of the irrigation pump; 3) teaching their children the newly acquired agricultural methods; and 4) training other community members not enrolled in Shamba Maisha. Control participants did not respond with any descriptions of confidence about future security.

#### Mechanism 1: Confidence in skills with enduring value

Some participants expressed confidence in skills learned through agricultural trainings that protected them against poor seasonal yields, such as crops ruined by rains. Participants expressed a perception that Shamba Maisha will change their life in the future:

“It (farming yields) will be very good and even the bananas the way I have planted them I see that they will also yield so much in future, because they have taught me how I am supposed to plant the bananas very well.”

She went on to say, when asked about her family’s future diet, a response that was given by multiple participants to express how the SM training has prepared them for the future,

“When I look at my family in the future, I see that we will be very ok, being that I am already in Shamba Maisha and know that it will help me here in future because if the rains reduce, I know that I will plant a lot of vegetables because I have been taught how to plant vegetables well, and even maize I see that I will be having a lot of maize.” (Female participant, intervention end, 28 years old)

#### Mechanism 2: Ownership of the irrigation pump

Ownership of the pump was protective against future unexpected weather events because participants expressed agency to teach others in their community how to use the pump to mitigate effects of droughts and inspiring others to invest in their own pump, thereby increasing the number of people in the community with capacity to produce food during extreme weather events.

A 44-year-old male participant described a community leader seeking advice about farming and irrigation and passing on knowledge to future generations:

“He could keep a pump in the house for future use and even after retirement could do the same thing as me. One of the people I am talking about is an official from a respected regional private school. He has been coming around inquiring about the pump and also to see how I work on the farm. He told me that although he is well off, his grandchildren may be able to use the pump some day and that it could be something that would help his family in the future.”

#### Mechanism 3: Teaching their children newly acquired farming skills

Plans to teach children how to farm with new methods and to use irrigation pump was an emergent theme expressed by some participants. This 44-year-old male participant described his confidence in his family using the irrigation pump in the future:

“Having the pump has changed our lives a lot because it’s a system that initially we never had but currently every community member would wish to have it. People even ask us where we got the pump. Personally, I think that it can bring positive changes in one’s life as it’s a good investment to those who own it. Such is the case with my family–if someday I am not around and one of them is able to use it, then they won’t have to worry a lot.”

#### Mechanism 4: Improvements in participants’ farmer identity among the community leads to expansion of Shamba Maisha training to other community members

An emergent theme related to farmer identity was described by one-fourth of participants. They explained how their improved crop yields changed their identity from low-producing farmers to high-producing farmers, causing community members to inquire about their new farming techniques, thus preparing the broader community against future weather or economic events. One 35-year-old female participant spoke to this point:

“The biggest change is in how we farm and that has made us be the points of consultation when the community members want to do their own farming. It is definitely (because of) Shamba Maisha through its trainings.”

Another participant explained how Shamba Maisha has worked to eliminate rejection of PLHIV from other people in the community who have stigmatized PLHIV:

“Shamba Maisha has already done that (eliminate rejection in the community). Other members of the community come to inquire how we are doing our farm work and they know that it is Shamba Maisha that has given us the skills that we are using.” (Female participant, intervention end, 30 years old)

Another participant attributed changes in community member perceptions of Shamba Maisha participants to the agricultural trainings which in turn influenced people to seek training from her:

“We were viewed that way (stigmatized), but now we are the ones who have vegetables and other people are coming to learn farming techniques from us. The pumps that we were given and the farm work we do help us exercise thereby, enabling us to go about our duties and the perception has changed.” (Female participant, intervention end, 35 years old)

This participant described how a community member expressed more confidence in Shamba Maisha farming techniques against future unexpected economic events, than a contracted formal sector job:

“Majority of those who wanted the pumps are not just HIV positive, but also people who have formal employment. When I asked one of them why someone who is working would need a pump, he told me that his contract could end at any time and after seeing how I was working on my farm, he saw the benefits.” (Male participant, intervention end, 44 years old)

He went on to describe a person who has learned from him:

“This farming method of Shamba Maisha… Yes, there are people who really envy it. There is also someone whom I have personally taught how it works and he has already started implementing it. There is also an old man who used to help me around the farm who learned by watching me.” (Male participant, intervention end, 44 years old)

### Challenges with intervention components

Some participants expressed frustration with the timing of their loan and irrigation pump distribution because they received the pump during the rainy season and were not able to use it until after repayment of the loan was due. In some cases people described feeling stress from the pressure to repay the loan. This participant describes both issues below.

“…the micro finance institution…which has also put a lot of pressure on us to repay the loan, this has really stressed us HIV positive people. They gave farm implements to use and this was a loan which they have given us a three months’ notice to repay… Of late it has been raining a lot so the crops could not grow well. Also the micro irrigation pump we had been given we have not been able to use as it does not make sense to irrigate the farm and increase the damage to the crops. So we are still storing the micro-irrigation pumps in our houses but the microfinance institution’s grace period for loan repayment is still three months. This matter is really stressing us…” (Male intervention participant, intervention end, 33 years old.)

Some participants expressed difficulty operating the irrigation pump alone explaining that a second person was required to pump the water while the participant held the hose to spray the crops. Participants described expending “a lot of energy” to operate the pump which in turn made them feel weak afterwards.

“…the machine I was given is too heavy for a woman to handle… The pipe needs to be directed by hand as someone pumps it…if my children are not around, I cannot do it alone, the machine just lies unused….If we could get one that is being stepped on (operated by feet) it would be better since the other one is heavy.” (Female intervention participant, intervention month 3, 48 years old.)

One participant described the limitations in who could use the pump and also needing to increase calorie consumption to operate the pump.

“This is our greatest problem. You cannot use the pump without eating; it requires a lot of energy…even if you take a heavy breakfast, after 30 minutes you must eat again to continue operating it… (Male participant, intervention month 3, 34 years old.)

Some participants had varying requests for improvements to the agricultural training such as more time with the trainers, additional information on seeds that would be most likely to survive local weather, as well as more information on how to choose what crops to grow based on supply and demand. One participant described crop losses with the seeds provided through the agricultural training and a failure to earn a profit from crops due to excess supply in the markets.

“The seeds we were given also did not also benefit us much since the when we planted them the heavy rains came, if there are heavy rains the seeds rot in the soil, some were swept away by the floods some, some germinated but did not multiply since the rain was too much and if you were lucky to have any that survived the supply in the market is so much that it cannot get any profits…” (Female participant, intervention month 3, 40 years old.)

Despite these challenges and recommendations for improvement, all of the above participants noted that they benefited from the intervention in terms of improved income, food security and well-being.

## Discussion

In this longitudinal, qualitative evaluation of an agriculture and finance intervention in Kenya, we found that intervention participants perceived considerable improvements in their farming practices, food security and nutrition, and protection from future unexpected economic and weather-related events, as compared to control participants. There were two primary mechanisms driving pathways to perceived improvements: the newly acquired farming skills and implements, including the irrigation pump, which led to improved yields, and income from high yielding farming practices used to supplement and diversify the diet and, in some cases, provide financial savings for the future. Participants from this study shared aspects of the intervention that could be improved including the irrigation pump’s design, the timing of the loan repayment, access to the agricultural trainers, and additional support to address weather shocks to crops. This helped the team to enhance the intervention prior to implementation of the larger cluster RCT (cRCT) which began in June 2016. The main change to the cRCT was improved agricultural training and changing to a higher volume treadle pump (MoneyMaker Max).

Farming is a complex and multifaceted occupation requiring knowledge across multiple domains including soil science, horticulture, crop utilization, and marketing. Most of these domains played a critical role in our study participants’ ability to convert their farming practices into ample food supplies for home consumption and additional income. The inclusion of these domains represents a common theme among agricultural development programs [43], however, this intervention creates a novel approach by using sustainable farming practices such as crop rotation to preserve soil nutrients and soil and water management to prevent erosion and lessen the effects of flooding, to address agricultural livelihood improvements within a health-affected population. This study elucidates the underlying pathways between an agricultural intervention and perceived improved outcomes in the setting of a chronic disease, and in the setting of worsening climate change.

Several key mechanisms were found among HIV-positive farmers, to create a framework for improved food security, nutrition, income, and protection for the future, besides changes in agricultural practices. Early in the study, participants experienced the benefits of diversification of their diets, noting improved tolerance of ART medications and feelings of improved energy, which enabled them to conduct their farm work. While this was not an intentional part of our training, it is important to note the beneficial effects this had on ongoing participation in agricultural work. This created a positive feedback loop reinforcing the benefits of implementing the agricultural training into participants’ farming practices. Another mechanism identified was the substantial increase in quantity of food produced, sometimes creating excess that went to waste after home consumption and market sales. This increase in yield quantity converted home gardeners to income-earning farmers and enabled market utilization in a variety of settings previously unavailable to participants. Participants’ ability to utilize the irrigation pump during periods of drought, further opened access to fresh produce markets driving up prices because of supply shortages and the increase in demand, and also mitigated impacts of climate-change induced drought.

Lastly, participants determined the impacts of the intervention on improved food security and nutrition extended beyond the study period, by passing on newly acquired knowledge of agricultural techniques to their children, other family, and community members, and because of their confidence in their ability to save excess income from crop sales. In addition, the irrigation pump was viewed as an asset beyond the study period and a mechanism for protection from future unexpected economic or weather-related events. These mechanisms help explain how increases in food security, decreases in ART side effects, decreases in competing demands for food and other resources led to improved ART adherence and clinic attendance in previously published findings from Shamba Maisha [41].

Some participants emphasized feelings of hope for their future, as previously reported, because of newly acquired knowledge they expected to use for the rest of their lives [40]. Their hope for the future extended to their children and broader communities. In this setting, where most people depend upon smallholder farming to supply much of their household food, it is common for community members to rely on one another during times of shortage. It is also common for community members to finance each other’s investments and work together to invest in farming resources that can be shared by groups. To this end, an important theme emerged indicating that Shamba Maisha was reaching beyond the participants, and into the community for people who wanted to improve their farming, food security and nutrition. Shamba Maisha participants expressed their motivations to share their knowledge to help others, and as protection for the entire community against future shortages of food.

The relationship between agriculture and food security in Sub-Saharan Africa (SSA) is a critical force for downstream nutrition, HIV outcomes, and other health metrics. To achieve the United Nations Sustainable Development Goal #2 by 2030, which aims to end hunger, achieve food security and improved nutrition, and promote sustainable agriculture, agricultural livelihood interventions are an important tool that can be used to improve nutrition and health. Shamba Maisha’s agricultural and finance intervention showed that addressing root causes of food insecurity in HIV-positive farmers could provide a pathway towards improved HIV outcomes through improvements in food security and nutrition. At the same time, addressing root causes of food insecurity brings populations currently dependent upon global aid, closer to self-sustaining livelihoods. Farmer training among populations susceptible to unexpected weather and economic events, can provide security against future risks of poverty, food insecurity and adverse health across the lifespan. The impacts of agricultural interventions for smallholder farmers living in regions affected by climate change, are likely to increase as weather shocks become more extreme.

Agricultural extension services are needed more broadly and with greater frequency to support smallholder farmers aiming to transition from subsistence farming to livelihood farming. This is important because over time, new problems arise related to maintenance or expansion of farming practices such as new pests that will require additional, ongoing agricultural training and consultation. Therefore, it is critical that governments prioritize these extension services to assist in breaking the feedback loop between food insecurity, poverty, and HIV in rural communities.

Multisectoral approaches that target food insecurity and poverty through improving nutrition and health are urgently needed. Since the majority of nutritionally vulnerable people in the world make their living and acquire food from smallholder farming, multisectoral agricultural and health interventions should comprise an important part of the solution.

This study indicates that by developing innovative, sustainable agriculture, food security and development interventions that focus on health targets, people can realize improvements in multiple areas of their life. These types of multisectoral interventions can systematically examine upstream causes of food insecurity and poor health, including poverty and environmental challenges such as climate change, all of which is necessary to achieve SDG targets.

## Limitations

Given the number of variables that can affect farming yields, the 12-month longitudinal study may have been too short in duration to understand the longevity of changes in farming practices. Agricultural training was limited to one trainer during the pilot study, keeping the intervention tightly controlled against variability expected when multiple people are providing agricultural training sessions. In real world settings, standardizing agricultural extension services requires significant training which is critical for reproducibility of study protocols [44–46]. Qualitative data could have some bias from participants under-reporting undesirable behaviors or over-reporting desirable behaviors or outcomes and recall bias could have occurred in those participants who were only interviewed at month 12 instead of at both 3 and 12-months, which could have excluded some undesirable behaviors or outcomes from those interviews [47]. However, we did compare 12-month only interviews with both controls and the longitudinal data and saturation was reached. Utilization, one of four dimensions of the United Nations Food and Agriculture Organization’s definition of food security, which includes non-food components such as clean water, sanitation, and other requirements to utilize food to attain a state of wellness, was not included in the training. We also did not focus on nutrition in the agricultural training, and this could have been useful for participants to better understand how certain foods could positively impact their health. Even though previously published data from our pilot study showed that food security scores were improved, including utilization components to the intervention could have improved food security further [48, 49]. Teaching participants how to utilize their crops to optimize nutritional and energy consumption as well as preserving during surplus yields, could prove beneficial in improving food security and nutritional status in both the short- and long-term.

## Conclusion

The Pilot Shamba Maisha study is one of a small cohort of theory-based, multisectoral interventions that incorporates an agricultural improvement model, financial training, and a well-established HIV care regimen. This allows for evaluation of how both livelihood and health requirements interrelate, which is critical for livelihood and health improvements to be sustained. More work is needed to understand how best to address food insecurity and poverty at a larger scale in smallholder farmer communities of PLHIV.

## Supporting information

S1 Checklist (PDF)
